# Demographic, clinical, and service-use characteristics related to the clinician’s recommendation to transition from child to adult mental health services

**DOI:** 10.1007/s00127-022-02238-6

**Published:** 2022-02-10

**Authors:** S. E. Gerritsen, L. S. van Bodegom, G. C. Dieleman, M. M. Overbeek, F. C. Verhulst, D. Wolke, D. Rizopoulos, R. Appleton, T. A. M. J. van Amelsvoort, C. Bodier Rethore, F. Bonnet-Brilhault, I. Charvin, D. Da Fonseca, N. Davidović, K. Dodig-Ćurković, A. Ferrari, F. Fiori, T. Franić, C. Gatherer, G. de Girolamo, N. Heaney, G. Hendrickx, R. Jardri, A. Kolozsvari, H. Lida-Pulik, K. Lievesley, J. Madan, M. Mastroianni, V. Maurice, F. McNicholas, R. Nacinovich, A. Parenti, M. Paul, D. Purper-Ouakil, L. Rivolta, V. de Roeck, F. Russet, M. C. Saam, I. Sagar-Ouriaghli, P. J. Santosh, A. Sartor, U. M. E. Schulze, P. Scocco, G. Signorini, S. P. Singh, J. Singh, M. Speranza, P. Stagi, P. Stagni, C. Street, P. Tah, E. Tanase, S. Tremmery, A. Tuffrey, H. Tuomainen, L. Walker, A. Wilson, A. Maras, for the Milestone Consortium, Laura Adams, Giovanni Allibrio, Marco Armando, Sonja Aslan, Nadia Baccanelli, Monica Balaudo, Fabia Bergamo, Angelo Bertani, Jo Berriman, Albert Boon, Karen Braamse, Ulrike Breuninger, Maura Buttiglione, Sarah Buttle, Aurélie Schandrin, Marco Cammarano, Alastair Canaway, Fortunata Cantini, Cristiano Cappellari, Marta Carenini, Giuseppe Carrà, Cecilia Ferrari, Krizia Chianura, Philippa Coleman, Annalisa Colonna, Patrizia Conese, Raffaella Costanzo, Claire Daffern, Marina Danckaerts, Andrea de Giacomo, Jean-Pierre Ermans, Alan Farmer, Jörg M. Fegert, Sabrina Ferrari, Giuliana Galea, Michela Gatta, Elisa Gheza, Giacomo Goglia, MariaRosa Grandetto, James Griffin, Flavia Micol Levi, Véronique Humbertclaude, Nicola Ingravallo, Roberta Invernizzi, Caoimhe Kelly, Meghan Killilea, James Kirwan, Catherine Klockaerts, Vlatka Kovač, Ashley Liew, Christel Lippens, Francesca Macchi, Lidia Manenti, Francesco Margari, Lucia Margari, Paola Martinelli, Leighton McFadden, Deny Menghini, Sarah Miller, Emiliano Monzani, Giorgia Morini, Todor Mutafov, Lesley O’Hara, Cristina Negrinotti, Emmanuel Nelis, Francesca Neri, Paulina Nikolova, Marzia Nossa, Maria Giulia Cataldo, Michele Noterdaeme, Francesca Operto, Vittoria Panaro, Adriana Pastore, Vinuthna Pemmaraju, Ann Pepermans, Maria Giuseppina Petruzzelli, Anna Presicci, Catherine Prigent, Francesco Rinaldi, Erika Riva, Anne Roekens, Ben Rogers, Pablo Ronzini, Vehbi Sakar, Selena Salvetti, Ottaviano Martinelli, Tanveer Sandhu, Renate Schepker, Marco Siviero, Michael Slowik, Courtney Smyth, Patrizia Conti, Maria Antonietta Spadone, Fabrizio Starace, Patrizia Stoppa, Lucia Tansini, Cecilia Toselli, Guido Trabucchi, Maria Tubito, Arno van Dam, Hanne van Gutschoven, Dirk van West, Fabio Vanni, Chiara Vannicola, Cristiana Varuzza, Pamela Varvara, Patrizia Ventura, Stefano Vicari, Stefania Vicini, Carolin von Bentzel, Philip Wells, Beata Williams, Marina Zabarella, Anna Zamboni, Edda Zanetti

**Affiliations:** 1 Department of Child and Adolescent Psychiatry and Psychology, Erasmus Medical Center, P.O. Box 2060, 3000 CB Rotterdam, The Netherlands; 2 Yulius Academy, Yulius Mental Health Organization, Dordrecht, The Netherlands; 3 Clinical Child and Family Studies, Vrije Universiteit Amsterdam, Amsterdam, The Netherlands; 4 Department of Clinical Medicine, University of Copenhagen, Copenhagen, Denmark; 5 Department of Psychology, University of Warwick, Coventry, UK; 6 Department of Biostatistics, Erasmus Medical Center, Rotterdam, The Netherlands; 7 NIHR Mental Health Policy Research Unit, Division of Psychiatry, University College London, London, UK; 8 Department of Psychiatry and Neuropsychology, University of Maastricht, Maastricht, The Netherlands; 9 Mondriaan Mental Health Care, Heerlen, The Netherlands; 10 Centre Hospitalier Universitaire de Tours, Tours, France; 11 Centre Hospitalier Universitaire de Marseille, Marseille, France; 12 University Hospital Split, Split, Croatia; 13 School of Medicine, University of Split, Split, Croatia; 14 Faculty for Dental Care and Health, Osijek, Croatia; 15 University Health Center Osijek, Osijek, Croatia; 16 Unit for Child and Adolescent Psychiatry, Osijek, Croatia; 17 IRCCS Istituto Centro San Giovanni di Dio Fatebenefratelli, Brescia, Italy; 18 DISM, ULSS 16, SOPROXI Onlus, Padua, Italy; 19 Department of Child and Adolescent Psychiatry, Institute of Psychiatry, Psychology and Neuroscience, Kings College London, London, UK; 20 Centre for Interventional Paediatric Psychopharmacology and Rare Diseases, South London and Maudsley NHS Foundation Trust, London, UK; 21 HealthTracker Ltd, Kent, UK; 22 Warwick Medical School, University of Warwick, Coventry, UK; 23 Department of Neurosciences, Centre for Clinical Psychiatry, KU Leuven, Leuven, Belgium; 24 Lille Neurosciences and Cognitions, Plasticity and Subjectivity Team, CURE Platform, Université de Lille, INSERM (U-1172), Fontan Hospital, CHU Lille, Lille, France; 25 CH Versailles, Versailles, France; 26 Warwick Clinical Trials Unit, Warwick Medical School, University of Warwick, Coventry, UK; 27 Centre Hospitalier Universitaire de Montpellier, Saint Eloi Hospital, Montpellier, France; 28 School of Medicine & Medical Science, University College Dublin, Dublin, Republic of Ireland; 29 Lucena CAMHS, SJOG, Dublin, Republic of Ireland; 30 Child and Adolescent Neuropsychiatry Unit, ASST Monza, Monza, Italy; 31 Università Degli Studi Milano Bicocca, Milan, Italy; 32 Centre Hospitalier Universitaire de Lille, Lille, France; 33 Coventry and Warwickshire Partnership NHS Trust, Coventry, UK; 34 INSERM, CESP U1018, PsyDev, University Paris Saclay, UVSQ, Versailles, France; 35 Psychiatric Epidemiology and Evaluation Unit, Saint John of God Clinical Research Center, Brescia, Italy; 36 Department of Mental Health, Psychiatry Unit, San Gerardo Hospital, Monza, Monza Brianza Italy; 37 Department of Neurosciences, KU Leuven, Leuven, Belgium; 38 Child and Youth Studies, Campus Social School, University Colleges Leuven Limburg, Heverlee, Belgium; 39 Department of Child and Adolescent Psychiatry/Psychotherapy, University of Ulm, Ulm, Germany; 40 Josefinum Augsburg, Klinik für Kinder- und Jugenspsychiatrie und Psychotherapie, Augsburg, Germany; 41 Department of Mental Health, ULSS 6 Euganea, Padua, Italy; 42 SOPROXI Onlus, Padua, Italy; 43 Service Universitaire de Psychiatrie de l’Enfant et de l’Adolescent, Centre Hospitalier de Versailles, Versailles, France; 44 Child and Adolescent Neuropsychiatry Unit, AUSL Modena, Modena, Italy; 45 Child and Adolescent Neuropsychiatry, Department of Mental Health, Modena, Italy; 46 Abteilung für Psychiatrie und Psychotherapie des Kindes-und Jugendalters Weissenau, ZfP Südwürttemberg, Ravensburg, Germany

**Keywords:** Child and adolescent mental health services, Adult mental health services, Young adults, Transition

## Abstract

**Purpose:**

The service configuration with distinct child and adolescent mental health services (CAMHS) and adult mental health services (AMHS) may be a barrier to continuity of care. Because of a lack of transition policy, CAMHS clinicians have to decide whether and when a young person should transition to AMHS. This study describes which characteristics are associated with the clinicians’ advice to continue treatment at AMHS.

**Methods:**

Demographic, family, clinical, treatment, and service-use characteristics of the MILESTONE cohort of 763 young people from 39 CAMHS in Europe were assessed using multi-informant and standardized assessment tools. Logistic mixed models were fitted to assess the relationship between these characteristics and clinicians’ transition recommendations.

**Results:**

Young people with higher clinician-rated severity of psychopathology scores, with self- and parent-reported need for ongoing treatment, with lower everyday functional skills and without self-reported psychotic experiences were more likely to be recommended to continue treatment. Among those who had been recommended to continue treatment, young people who used psychotropic medication, who had been in CAMHS for more than a year, and for whom appropriate AMHS were available were more likely to be recommended to continue treatment at AMHS. Young people whose parents indicated a need for ongoing treatment were more likely to be recommended to stay in CAMHS.

**Conclusion:**

Although the decision regarding continuity of treatment was mostly determined by a small set of clinical characteristics, the recommendation to continue treatment at AMHS was mostly affected by service-use related characteristics, such as the availability of appropriate services.

**Supplementary Information:**

The online version contains supplementary material available at 10.1007/s00127-022-02238-6.

## Introduction

Mental health services for children and adolescents will usually provide care until the young person is aged 16–19 years. Some argue that the provision of distinct child and adolescent mental health services (CAMHS) and adult mental health services (AMHS) may hamper the continuity of care [1, 2] and that this discontinuity may adversely affect the mental health of young people. Indeed, previous research indicates 30% to 84% of young people experience a discontinuity of care after reaching the upper age limit of their CAMHS [3–5], yet no studies to date have investigated how a discontinuity of care may affect the mental health of young people. Additionally, previous studies often do not clearly indicate whether or not the discontinuity of care is in accordance with the recommendation from the CAMHS clinician, i.e., that further mental health care was not required. To provide insight in reasons for this discontinuity, it is important to disentangle the different steps in the process of transition from CAMHS to AMHS.

Once young people reach the upper age limit of their CAMHS, clinicians need to advise patients and their parents about the type of care, if any, that will be needed going forwards to ensure optimal mental health. First, the clinicians’ recommendations address any need for continued mental health care. Second, if continuation of mental health care is deemed necessary, the clinician needs to decide where such care should be provided. In certain circumstances, this might include continuing at CAMHS for a short period to conclude treatment, or alternatively, transfer to AMHS or to another type of mental health service. The clinician’s recommendation regarding continuity of care is the first step in the process of transition and may have a significant impact on the young person’s outcomes. Therefore, our study focusses on the clinician’s transition recommendation and aims to describe which factors are associated with this recommendation.

Several studies have tested factors associated with referral to AMHS after adolescents reached the upper age limit of their mental health services. The results of these studies are consistent in showing that a clinical classification of a psychotic or personality disorder is associated with a greater likelihood of referral to adult mental health care [4, 6, 7], but are inconclusive with regard to demographic and family characteristics and treatment and service-use related characteristics, specifically length of stay in CAMHS and psychotropic medication use [4, 6, 7]. Comparison of the results of existing studies is hampered by variations in methodology, such as whether bivariate or multivariate analyses are used, and variations in the groups that were compared: most studies compared young people who were referred or transitioned to AMHS to young people who did not [4, 5, 7]. Other studies described young people who were referred to AMHS or transitioned compared to those who were discharged [8] or those who stayed in CAMHS [9]. Other indicators of severity of psychopathology have not previously been studied in relation to referral such as the clinician’s rating of severity of psychopathology, self- and parent-reported problem levels, psychotic experiences, suicidality, daily functional skills, self- and parent-reported need for ongoing treatment, inpatient service use, and visits to the accident and emergency department.

This European study used multi-informant standardized assessment to determine which demographic, family, clinical, treatment, and service-use-related characteristics are associated with clinicians’ transition recommendation to continue care in general, and the recommendation to continue care at AMHS specifically, using multivariate analyses.

## Methods

### Study design and participants

**Fig. 1:**
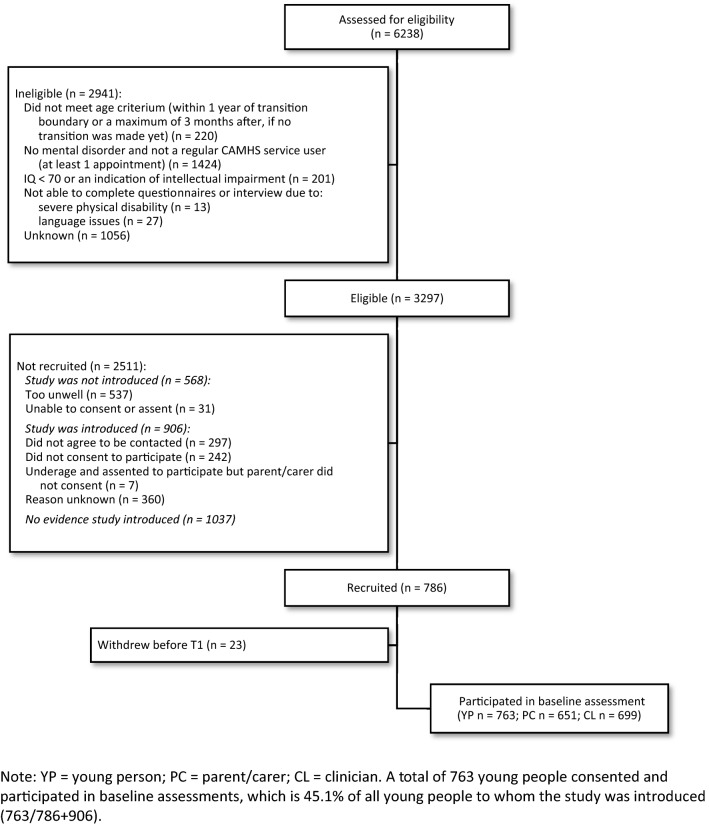
CONSORT flow diagram of participants

This study was carried out on the MILESTONE cohort [10, 11]. A total of 786 young people were recruited from 39 CAMHS in Europe (Belgium, Croatia, France, Germany, Ireland, Italy, The Netherlands, and The United Kingdom) between October 2015 and December 2016 (see Fig. 1). Eligible young people were receiving care in CAMHS and within a year of reaching the upper age limit of that CAMHS (or three months after, if still in CAMHS). A total of 6238 young people had a patient record at one of 39 CAMHS and met the age criterion. They were assessed further for eligibility and inclusion criteria by care coordinators and clinicians. A total of 2941 young people were considered ineligible, of which 220 young people no longer met the age criterion and 1424 no longer visited the CAMHS at the time they could be informed about the study. Care coordinators and clinicians introduced the study to 1692 young people, after which young people indicated whether they consented to being contacted by the research team. Due to medical ethical reasons, our research team was not allowed to contact young people to inform them about the study directly. Of all young people to whom the study was introduced (*n* = 1692), 763 young people (45.1%) were recruited to the MILESTONE cohort.

Country-specific consent procedures were followed, according to national laws and medical ethical regulations. A parent and a CAMHS clinician (a mental health professional responsible for, or coordinating, the care for the young person) were also asked to participate in the study. A total of 763 young people consented to participate and completed baseline assessments. Gerritsen et al. [11] provide a detailed description of the recruitment process and cohort characteristics. The study protocol was approved (ISRCTN83240263; NCT03013595) by the UK National Research Ethics Service Committee West Midlands—South Birmingham (15/WM/0052) and ethics boards in participating countries.

### Procedure

Baseline assessments were conducted after consent was provided. Young people and their parents completed interviews and questionnaires during assessments which took place at the local CAMHS, at home or over the phone. Questionnaires were administered online via the HealthTracker^TM^ platform; paper copies were only used if HealthTracker could not be accessed. Clinicians completed questionnaires and/or medical records were accessed to provide clinical information on the young person. Data collected at baseline included information on demographic, family, clinical, treatment, and service-use characteristics as well as on transition recommendations.

### Measures

#### Demographic, family, clinical, treatment, and service-use characteristics

Demographic and family characteristics included living situation, education/employment, and psychopathology in biological parents. Clinical characteristics included self- and parent-reported emotional/behavioural problems (collectively referred to as ‘problem levels’), clinical classifications, clinician-rated severity of psychopathology, self-reported suicidal thoughts/behaviours or self-harm, self-reported psychotic experiences, and parent-reported everyday functional skills. Treatment and service-use characteristics included self-reported inpatient psychiatric service use, accident and emergency department service use, psychotropic medication use, self-reported length of CAMHS use, the availability of appropriate AMHS according to the clinician (i.e., a local AMHS service with the skills/resources to treat the young person's condition), and the need for ongoing treatment irrespective of the type of care or service indicated by young people and parents. All measures described in this paper were administered at baseline and are listed in Table 1.

**Table 1:** Measures

Construct	Informant (method)	Instruments	Description	Psychometrics	Scoring
Demographic and family characteristics					
Socio-demographic characteristics	YP (I)	Socio-demographic interview	Assessing socio-demographic variables, such as living situation, education/employment	The socio-demographic interview was largely based on the Client Socio-demographic and Service Receipt Inventory EU version (CSSRI-EU [32]; Psychometric properties of CSSRI-EU for assessing socio-demographic variables are not available, but the instrument has been validated in a large European study on mental health: EPSILON [32]	All responses were categorical Living situation was categorized as (1) with parents/carers (one or both biological parents, with or without partner, foster or adoptive parents) or (2) not living with parents/carers (including living with partner, roommates, independently, living residentially or ‘other’ Education/occupation was categorized as (1) in school or working (including voluntary employment) or (2) not in school or working
Family characteristics	PC (I)	Socio-demographic interview (PC-version)	Highest level of PC education of either parent (‘What is your highest completed level of education?”) and (history of) psychopathology in biological parents (“Were you ever examined or treated for mental, developmental, language, speech or learning problems?”) was assessed in the socio-demographic interview	The item on level of education came from the CSSRI-EU (see psychometrics for ‘socio-demographic characteristics’)	Level of PC education was categorized as (1) primary or secondary/vocational or (2) university. If information on the level of education of both PCs was available, we used the level of the PC with the highest educational level Psychopathology in biological parents was categorized as (1) psychopathology in one or both biological parents or (2) no psychopathology. The response was set to missing if the respondent was not a biological parent
Clinical characteristics					
Clinical classifications	CL (I)	Clinical classifications	Official clinical classifications registered in the medical records (or, if no official diagnosis was registered: the preliminary/working diagnosis registered)	The classification was based on the Diagnostic and Statistical Manual of Mental Disorders, version IV or 5 and the International Classification of Diseases, version 10	Clinical classifications are dummy coded and indicate presence or absence of a specific clinical diagnosis or category. Diagnosis categories were collapsed to three broad categories: emotional disorders (depressive, anxiety, eating, trauma, obsessive–compulsive or somatic disorders); behavioural/neurodevelopmental disorders (ADHD, ASD or CD), severe mental illnesses (bipolar, personality and schizophrenia spectrum disorders)
Emotional and Behavioural Problems	YP, PC (OQ)	Youth Self-Report (YSR) Adult Self-Report (ASR) Child Behaviour Checklist (CBCL) Adult Behaviour Checklist (ABCL)	YP (YSR/ASR) and PC reported (CBCL/ABCL) emotional and behavioural problems in the last 6 months in versions for YP under (YSR/CBCL) or over (ASR/ABCL) 18 years old	The Achenbach System of Empirically Based Assessment [33, 34] (ASEBA) instruments have been used extensively in different contexts and have shown excellent psychometric properties	Mean item scores (ranging 0 to 2) were calculated allowing us to combine YSR and ASR scores to compute total self-reported problem scores and total CBCL/ABCL scores to compute parent-reported problem scores. Higher scores indicate more internalizing/externalizing problems
Clinician rated severity of psychopathology	CL (I)	Clinical Global Impression – Severity scale (CGI-S)	CL rated severity of psychopathology over the last week relative to other patients with similar problems	The CGI-S [35] is extensively used in psychiatric research [36] and has proven useful in predicting suicidal ideation and behaviours [37]	Single score measuring severity on a 7-point scale from ‘not at all ill’ (score = 1) to ‘among the most extremely ill’ (score = 7)
Suicidal thoughts/behaviours or self-harm	YP (OQ)	Suicidal thoughts/behaviours or self-harm was assessed with 2 items in the Transition Readiness and Appropriateness Measure (TRAM), developed by the MILESTONE consortium	Self-harming behaviour was assessed with the item “I have injured myself on purpose without intending to kill myself by cutting, scratching, burning, overdosing on pills, swallowing harmful objects/liquids or other methods” and suicidal thoughts & behaviours with “I have suicidal thoughts, wish I was dead, imagine how I would kill myself, and/or have attempted to end my own life.”	The TRAM has been established to be a reliable instrument for assessing transition readiness and appropriateness. The ‘suicidal thoughts’ and ‘self-harming behaviour’ items had high ‘risk’-factor loading scores, indicating its relevance to the preparedness of transition [12]	No ‘suicidal thoughts/behaviours or self-harm’ was scored when the respondent indicated the young person had ‘not experienced’ or ‘rarely experienced’ self-harming behaviour or suicidal thoughts and behaviours. If a response of ‘sometimes’, ‘often’, ‘most of the time’, or ‘all of the time’ was given to either the self-harming or suicidal-thoughts-item, the variable ‘suicidal thoughts/behaviours or self-harm’ was scored as ‘yes’
Psychotic experiences	YP (OQ)	Development and Well-Being Assessment (DAWBA)	DAWBA assesses a range of psychiatric diagnoses through structured sections of the online questionnaire, among which psychotic experiences (to identify whether the young person has (had) psychosis). The open sections of the DAWBA were omitted to limit the burden on the participants and to standardize the classification procedure	The DAWBA psychotic experiences section proved valuable as a screening tool in the youth general population (it has not yet been validated in a clinical sample) [38]	Respondents indicated whether the young person ever experiences a range of psychotic experiences, with response options ‘no’, ‘a little’, and ‘a lot’. The total number of a total of 10 experiences the young person experiences (either a little or a lot) was calculated. Because the distribution of the number of experiences was zero-inflated, the variable was dichotomized. Having no or 1 psychotic experience(s) was coded as ‘0’, having had 2 or more experiences was categorized as 1
Everyday functional skills	PC (OQ)	Specific Levels of Functioning (SLOF)	Assesses YP’s everyday functional skills. It “emphasizes patient's current functioning and observable behaviour, as opposed to inferred mental or emotional states” [39]	The SLOF domains have acceptable internal consistencies (except for a Cronbach’s alpha of .55 for physical functioning) and good concurrent validity [40]	Average everyday functional skill-scores ranged from 1 to 5 on 6 domains: physical functioning, personal care, interpersonal relationships, social acceptability, activities and work skills, with higher scores indicating more everyday functional skills. A total score was computed to reflect overall everyday functional skills
Treatment and service-use related characteristics					
Medical history (length of CAMHS use)	YP (I)	Socio-demographic interview	Items on medical history (including length of CAMHS use) were added to the CSSRI-EU in the socio-demographic interview		Length of CAMHS use was categorized as (1) less than one year, (2) one to five years, or (3) more than five years
Inpatient psychiatric, accident and emergency, and medication use	YP (OQ)	CSSRI-EU (amended for use in a psychiatric setting)	Assesses inpatient and outpatient service use over the last 6 months in different settings (hospital, community and informal) and medication use over the last 6 months	The CSSRI-EU was found to be effective in tracing patterns of service use in an international population and made comparisons between different countries possible [32]	Dichotomous service-use scores over different service-use types, such as the accident & emergency department and inpatient psychiatric service use Young people provided information on medication use in open text fields
Availability of appropriate AMHS	CL (OQ)	TRAM	Availability of appropriate AMHS was assessed with the item “I am confident that there is a local AMHS service with the skills/resources to treat the young person's condition/s.”	The TRAM has been established to be a reliable instrument for assessing transition readiness and appropriateness [12]	The responses were scored as ‘strongly agree’ (2) to ‘strongly disagree’ (-2)
Need for ongoing treatment	YP, PC, CL (OQ)	TRAM	Need for ongoing treatment was assessed with the following item: “Does the young person require ongoing treatment to control their symptoms?”	See psychometric properties of the TRAM reported for ‘availability of appropriate AMHS’	Response categories were yes/no
Transition recommendation	CL (OQ)	TRAM	Transition recommendations were assessed with the item “What type of care do you consider most appropriate (and possible within your health care system) for the young person?”	See psychometric properties of the TRAM reported for ‘availability of appropriate AMHS’	The transition recommendation was dichotomised. First, either indicating a recommendation for continuity of treatment within a specialist mental healthcare setting (‘continue treatment at current CAMHS service’, ‘continue treatment by other mental health services’, or ‘transition to AMHS’) or ‘discontinuity’ (‘discharge’ or ‘discharge to the GP’). Secondly, we created a dichotomous variable to indicate whether continued care was recommended in CAMHS (‘continue treatment at CAMHS’) or AMHS (‘transition to AMHS’)

*YP* young person, *PC* parent/carer, *CL* clinician, I interview, *OQ* online questionnaire

#### Transition recommendations

Transition recommendations by the clinician were assessed using the Transition Readiness and Appropriateness Measure [12] (TRAM; see Table 1). To study characteristics associated with continued care in general and a recommendation to continue care at AMHS specifically, the transition recommendation was dichotomised in two ways. The first indicated a recommendation for continuity of treatment within a mental healthcare setting (if the clinician indicated it was most appropriate to ‘continue treatment at current CAMHS service’, ‘continue treatment by other mental health services’ or ‘transition to AMHS’) or discontinuity of treatment (if the clinician indicated it was most appropriate to ‘discharge’ or ‘discharge to the general practitioner’). Second, if the recommendation was to continue treatment, response categories were collapsed to indicate whether continued care was recommended in CAMHS (‘continue treatment at current CAMHS service’ or ‘continue treatment at other CAMHS’) or AMHS (‘transition to AMHS’).

#### Covariates

Gender, highest level of parental education, and country were reported and included as covariates in the analyses to account for potential confounding by these characteristics.

### Statistical analyses

Details on how the different measures were scored and applied in analyses are described in Table 1.

First, we assessed which demographic, family, clinical, treatment, and service-use-related characteristics of young people were associated with the clinician’s treatment recommendations, by fitting logistic mixed models. Demographic, family, clinical, treatment, and service-use related characteristics were included as independent variables, and the clinician’s recommendation was included as the dependent variable, dichotomized as needing continued mental health treatment versus not needing that treatment according to the clinician. Second, among young people who were recommended to continue mental health treatment, we used logistic mixed models to assess which demographic, family, clinical, treatment, and service-use-related characteristics were associated with being recommended to continue treatment at AMHS (i.e., to transition) versus CAMHS.

To account for potential confounding, all analyses were multivariate, and gender, parents’ highest completed level of education, and country were added as covariates. ‘Omnibus-tests’ were conducted to test whether adding non-linear effects (cubic splines) and/or interactions between clinical classifications and gender need for ongoing treatment and length of CAMHS use improved model fit. If an omnibus test indicated interactions and/or non-linear effects significantly contributed to an improved model fit, additional analyses were conducted to assess which specific effects improved the model.

Model fit was assessed by comparing the fit of the final model to a covariate only model with a likelihood ratio test. Pooled odds ratios and corresponding 95% confidence intervals were reported. The significance level was set at α = 0.05. Multicollinearity was not present, indicated by a maximum squared adjusted generalized variance inflation factor (GVIF^(1/(2*Df)), comparable to VIF) under 2. All statistical analyses were performed in R [13]. All models were fitted with site (CAMHS) as the only level and random intercepts (applying a likelihood estimator), using lme4 [14].

We assumed that data were missing at random (MAR), as we hypothesized that the missingness of an observation depended only on observed values. Previous analyses [11] support this assumption, as the missingness in data from parent-reported measures, which had largest proportion of missingness, was dependent on the observed values of self-reported and clinician-reported problem levels. To account for missing data under the MAR assumption, multiple imputation was applied on all variables included in the analyses before mixed models were fitted (accounting for clustering of the data; using mice [15] and miceadds [16]). The data were imputed using all variables included in the analyses described in this manuscript as predictor variables to estimate missing values. The variable ‘site’ (indicating the CAMHS in which the young person was recruited) was used as a cluster variable. The data were imputed with the default method in mice, after which density plots and trace lines were used to inspect the imputations and convergence, respectively. Following these inspections, the method of imputation was changed per variable, to see whether other methods improved the imputation. A total of 50 imputations were conducted with 20 iterations.

## Results

A total of 763 young people were recruited to the MILESTONE cohort. Their mean age was 17.5 years (SD = 0.59; ranging from 15.2 to 19.6). A total of 458 young people (60.0%) identified as female and 305 (40%) identified as male.

### Clinician recommendations

Clinicians recommended continuation of treatment within MHS for 460 (60.3%) of young people. Of those who were recommended to continue treatment, 52.4% (*n* = 241) were advised to stay in CAMHS, 32.4% (*n* = 149) to transition and continue treatment at AMHS, and 15.2% (*n* = 70) were recommended to continue treatment in ‘other’ MHS (not specifically CAMHS or AMHS). Clinicians recommended discharge or referral to the GP for 180 young people (23.6%). Information on the clinician’s recommendation was missing for 123 (16.1%).

### Characteristics associated with the clinician recommendation to continue treatment within mental health services

Table 2 shows descriptive characteristics of 640 young people without missing data on the clinician’s recommendation, as well as the results from the first analysis, conducted on imputed data (*n* = 763): the demographic, family, clinical, treatment, and service-use characteristics associated with the recommendation to continue care in MHS (CAMHS, AMHS, or other MHS) or to discontinue (referral to general practitioner or discharge). The model with the best model fit included a natural cubic spline for the severity of psychopathology score and did not include interactions, and predicted a recommendation of continued treatment significantly better than the covariate only model (*p* < 0.001).

**Table 2:** Demographic and family, clinical, and treatment and service characteristics in relation to transition recommendations regarding continuity of care (descriptives and model summary)

	Characteristics (original non-imputed data)			Model summary (on imputed data)*		
	*n*	Disc. (*n* = 180)	Cont. (*n* = 460)	OR	95% CI	
Demographic and family characteristics						
Living situation (*n* (%))	716					
Not living with parents/carers		8 (4.4)	47 (10.2)			
Living with parents/carers		171 (95.0)	396 (86.1)	0.74	0.28	1.99
Missing		1 (0.6)	17 (3.7)			
Education/employment (*n* (%))	713					
Not in school or working		7 (3.9)	41 (8.9)			
In school or working		170 (94.4)	401 (87.2)	0.69	0.22	2.12
Missing		3 (1.7)	18 (3.9)			
Psychopathology in biological parents (n (%))	545					
No psychopathology		96 (53.3)	225 (48.9)			
Psychopathology in one or both biological parents		47 (26.1)	121 (26.3)	1.32	0.70	2.48
Missing		37 (20.6)	114 (24.8)			
Clinical characteristics						
Self-reported total emotional/ behavioural problems (mean (SD))	683	0.50 (0.27)	0.58 (0.28)	1.17	0.30	4.55
Parent/carer-reported total emotional/ behavioural problems (mean (SD))	572	0.31 (0.21)	0.42 (0.24)	2.30	0.46	11.65
Clinical classifications (*n* (%))	734					
Emotional disorder^1^		107 (59.4)	284 (61.7)	1.24	0.66	2.33
Behavioural/neurodevelopmental disorder^2^		54 (30.0)	170 (37.0)	1.52	0.73	3.16
Severe mental illness^3^		8 (4.4)	77 (16.7)	2.42	0.89	6.58
Clinician rated severity of psychopathology (mean (SD))^4^	640	2.26 (1.08)	3.89 (1.24)			
ns (CGIS, 3)1				**36.02**	**9.86**	**131.65**^**#**^
ns (CGIS, 3)2				**171.75**	**17.79**	**1657.97**
ns (CGIS, 3)3				**19.91**	**1.27**	**310.97**
Suicidal thoughts/behaviours or self-harm (*n* (%))	626					
None		117 (65.0)	230 (50.0)			
Suicidal thoughts/behaviours or self-harm		60 (33.3)	210 (45.7)	1.86	0.92	3.75
Missing		3 (1.7)	20 (4.3)			
Psychotic experiences^5^ (*n* (%))						
0 or 1 experience(s)		104 (57.8)	274 (59.6)			
2–16 experiences		37 (20.6)	99 (21.5)	**0.29**	**0.13**	**0.62**
Missing		39 (21.7)	87 (18.9)			
Everyday functional skills (mean (SD))	577	4.58 [4.33, 4.81]	4.40 [4.05, 4.67]	**0.39**	**0.17**	**0.88**
Treatment and service use						
Inpatient psychiatric service use (*n* (%))	666					
No		164 (91.1)	358 (77.8)			
Yes		1 (0.6)	56 (12.2)	1.77	0.39	7.96
Missing		15 (8.3)	46 (10.0)			
Accident and emergency department service use (*n* (%))	666					
No		143 (79.4)	362 (78.7)			
Yes		22 (12.2)	52 (11.3)	0.73	0.32	1.66
Missing		15 (8.3)	46 (10.0)			
Psychotropic medication use (*n* (%))	666					
No		84 (46.7)	152 (33.0)			
Yes		81 (45.0)	262 (57.0)	1.22	0.66	2.25
Missing		15 (8.3)	46 (10.0)			
Length of CAMHS use^6^ (*n* (%))	703					
< 1 yr		39 (21.7)	123 (26.7)			
1–5 yrs		88 (48.9)	207 (45.0)	0.80	0.41	1.55
> 5 yrs		51 (28.3)	104 (22.6)	0.51	0.23	1.17
Missing		2 (1.1)	26 (5.7)			
Availability of appropriate AMHS (mean (SD))	640	0.28 (1.16)	0.30 (1.15)	1.14	0.89	1.45
YP indicating need for ongoing treatment (*n* (%))	716					
No		106 (58.9)	139 (30.2)			
Yes		71 (39.4)	301 (65.4)	**1.91**	**1.08**	**3.38**
Missing		3 (1.7)	20 (4.3)			
PC indicating need for ongoing treatment (*n* (%))	579					
No		81 (45.0)	88 (19.1)			
Yes		69 (38.3)	276 (60.0)	**2.44**	**1.26**	**4.74**
Missing		30 (16.7)	96 (20.9)			

Logistic mixed model was fitted with site as the only level and random intercepts (applying a likelihood estimator), displaying odds of continued treatment within MHS recommended versus with no continued treatment within MHS recommended as the reference group. Gender, parental educational level and country were added as covariates

*YP* young person, *PC* parent/carer, *Cont.* recommendation to continue treatment, *Disc*. recommendation to discontinue treatment

^1^Combination of depressive, anxiety, eating, trauma, obsessive–compulsive, and somatic disorders

^2^Combination of ADHD, ASD and CD

^3^Combination of bipolar, personality and schizophrenia spectrum disorders

^4^The effect of clinician-rated severity of psychopathology was non-linear, with a natural cubic spline. The effect was strongest for the third spline (ns(CGIS, 3)3) indicating the slope of the effect was steepest around scores 3 and 4

^5^Reference category is 0 or 1 psychotic experience(s)

^6^Reference category is less than 1 year

*n changes depending per imputed dataset

^#^The confidence intervals for splines are large, but this is not uncommon. As the coefficients for splines do not have a very direct interpretation, the effects of the splines of clinician-rated psychopathology are visually presented in the effect plot in the supplementary material

A higher severity of psychopathology score significantly predicted a recommendation for continued treatment within MHS. Self- and parent-reported emotional/behavioural problems were not associated with the clinician’s recommendation to continue treatment, but a young person or parent indicating a need for ongoing treatment did increase the odds of the clinician recommending continuation of treatment within MHS by more than 2. A clinical classification of a severe mental disorder or self-reported suicidal thoughts/behaviours or self-harm were not associated with the clinician’s recommendation. Additionally, young people who had more than one psychotic experience had 71% decrease in the odds of being recommended to continue treatment. Young people with more everyday functional skills were 2.5 times less likely to be recommended to continue treatment. Demographic and family characteristics such as having a job, attending school, or the living situation, were not associated with the clinician’s recommendation.

#### Unplanned explorative analyses on psychotic experiences

As the negative association between self-reported psychotic experiences and a recommendation to continue treatment was surprising, we conducted additional explorative bivariate analyses (Welch two-sample t tests) to gain insight in the differences between young people with self-reported psychotic experiences who were recommended to continue treatment and those who were not. We found that young people who reported psychotic experiences but were not recommended to continue treatment had lower clinician-rated severity of psychopathology scores (*t*(77.962) = − 8.989; *p* < 0.001) and lower research-assistant rated scores on the HoNOSCA ‘hallucinations and delusions’ domain (*t*(95.366) = − 2.877; *p* = 0.005), than young people who reported psychotic experiences who were recommended to continue treatment (these analyses were conducted on observed data). Additionally, young people who reported psychotic experiences and were recommended to continue treatment were more likely to have a clinical classification of a severe mental disorder (*t*(122.11) = − 3.873; *p* < 0.001) than young people who reported psychotic experiences, but were not recommended to continue treatment. There were no differences between these groups with regard to behavioural/neurodevelopmental disorders (*t*(64.078) = 0.012; *p* = 0.991) and emotional disorders (*t*(64.966) = 0.177; *p* = 0.860).

### Characteristics associated with the transition recommendation to continue treatment at AMHS

Among those for whom continued treatment in MHS was recommended, a differentiation was made between those who were recommended to continue treatment within CAMHS (*n* = 241) versus those who were recommended to transition to AMHS (*n* = 249). Table 3 shows demographic, family, clinical, treatment, and service-use characteristics of both groups. To assess how these characteristics of young people at baseline were associated with transition decisions, a model predicting the clinician’s recommendation for continued treatment at AMHS was established. The model with the best model fit did not include interactions or non-linear effects and predicted a recommendation to AMHS significantly better than the covariate only model (*p* = 0.004). Odds ratios and associated confidence intervals for this model are presented in Table 3.

**Table 3:** Demographic and family, clinical, and treatment and service characteristics in relation to transition recommendations regarding continued treatment in CAMHS versus AMHS (descriptives and model summary)

	*n*	Characteristics (original non-imputed data)		Model summary (on imputed data)*		
		CAMHS (*n* = 241)	AMHS (*n* = 149)	OR	95% CI	
Demographic and family characteristics						
Living situation (*n* (%))	716					
Not living with parents/carers		27 (11.2)	14 (9.4)			
Living with parents/carers		210 (87.1)	131 (87.9)	1.15	0.45	2.96
Missing		4 (1.7)	4 (2.7)			
Education/employment (*n* (%))	713					
Not in school or working		20 (8.3)	15 (10.1)			
In school or working		215 (89.2)	130 (87.2)	0.95	0.34	2.69
Missing		6 (2.5)	4 (2.7)			
Psychopathology in biological parents (*n* (%))	545					
No psychopathology		125 (51.9)	67 (45.0)			
Psychopathology in one or both biological parents		62 (25.7)	47 (31.5)	0.94	0.48	1.84
Missing		54 (22.4)	35 (23.5)			
Clinical characteristics						
Self-reported total emotional/ behavioural problems (mean (SD))	683	0.56 (0.26)	0.60 (0.30)	0.75	0.20	2.80
Parent/carer-reported total emotional/ behavioural problems (mean (SD))	572	0.38 (0.22)	0.47 (0.25)	4.59	0.80	26.4
Clinical classifications (*n* (%))	734					
Emotional disorder^1^		154 (63.9)	91 (61.1)	1.33	0.67	2.64
Behavioural/neuro- developmental disorder^2^		82 (34.0)	63 (42.3)	1.63	0.74	3.60
Severe mental illness^3^		38 (15.8)	27 (18.1)	1.59	0.70	3.60
Clinician rated severity of psychopathology (mean (SD))	640	3.84 (1.27)	3.94 (1.17)	1.27	0.98	1.65
Suicidal thoughts/behaviours or self-harm (*n* (%))	626					
None		128 (53.1)	73 (49.0)			
Suicidal thoughts/behaviours or self-harm		108 (44.8)	67 (45.0)	1.12	0.56	2.22
Missing		5 (2.1)	9 (6.0)			
Psychotic experiences^4^ (*n* (%))						
0 or 1 experience(s)		152 (63.1)	84 (56.4)			
2–16 experiences		49 (20.3)	35 (23.5)	1.29	0.59	2.78
Missing		40 (16.6)	30 (20.1)			
Everyday functional skills (mean (SD))	577	4.42 [4.14, 4.67]	4.27 [3.84, 4.65]	1.15	0.51	2.57
Treatment/service-use						
Inpatient psychiatric service use (*n* (%))	666					
No		196 (81.3)	110 (73.8)			
Yes		26 (10.8)	21 (14.1)	1.96	0.77	4.97
Missing		19 (7.9)	18 (12.1)			
Accident and emergency department service use (*n* (%))	666					
No		194 (80.5)	112 (75.2)			
Yes		28 (11.6)	19 (12.8)	1.14	0.44	2.95
Missing		19 (7.9)	18 (12.1)			
Psychotropic medication use (*n* (%))	666					
No		98 (40.7)	36 (24.2)			
Yes		124 (51.5)	95 (63.8)	**2.20**	**1.09**	**4.42**
Missing		19 (7.9)	18 (12.1)			
Length of CAMHS use^5^ (*n* (%))	703					
< 1 yr		85 (35.3)	23 (15.4)			
1–5 yrs		106 (44.0)	74 (49.7)	**2.01**	**1.02**	**3.98**
> 5 yrs		41 (17.0)	46 (30.9)	**3.11**	**1.23**	**7.89**
Missing		9 (3.7)	6 (4.0)			
Availability of appropriate AMHS (mean (SD))	640	0.12 (1.16)	0.57 (1.08)	**1.63**	**1.25**	**2.13**
YP indicating need for ongoing treatment (*n* (%))	716					
No		75 (31.1)	47 (31.5)			
Yes		161 (66.8)	93 (62.4)	0.75	0.39	1.45
Missing		5 (2.1)	9 (6.0)			
PC indicating need for ongoing treatment (*n* (%))	579					
No		41 (17.0)	35 (23.5)			
Yes		154 (63.9)	86 (57.7)	**0.34**	**0.16**	**0.73**
Missing		46 (19.1)	28 (18.8)			

Logistic mixed model was fitted with site as the only level and random intercepts (applying a likelihood estimator), displaying odds of continued treatment at AMHS recommended versus with continued treatment within CAMHS recommended as the reference group. Gender, parental educational level and country were added as covariates

*YP* young person, *PC* parent/carer

^1^Combination of depressive, anxiety, eating, trauma, obsessive–compulsive, and somatic disorders

^2^Combination of ADHD, ASD, and CD

^3^Combination of bipolar, personality, and schizophrenia spectrum disorders

^4^Reference category is 0 or 1 psychotic experience(s)

^5^Reference category is less than 1 year

*n changes depending per imputed dataset

Neither clinician-rated psychopathology, nor self- or parent-reported emotional/behavioural problems were associated with the clinician’s recommendation to transition to AMHS. The odds of the young person being recommended to transition to AMHS decreased by 66% when parents indicated a need for ongoing treatment, or alternatively: they were more likely to be recommended to stay in CAMHS. Having a severe mental disorder, self-reported suicidal thoughts/behaviours or self-harm, or self-reported psychotic experiences were not associated with the recommendation to transition to AMHS, nor were everyday functional skills, working or being in school and living independently. Medication use emerged as an important factor—young people who used psychotropic medication had over twice greater odds of being recommended to transition to AMHS. The length of time in CAMHS was also important: for young people who had been in CAMHS treatment for more than 1 year, the odds of being recommended to transition to AMHS doubled compared to young people who had been in treatment at CAMHS for less than a year. Having been at CAMHS for more than 5 years tripled the odds of being recommended to transition to AMHS compared to those in care less than a year. The clinician indicating appropriate AMHS were available for treatment of the young person increased the odds by 63%.

## Discussion

This study describes associations between demographic, family, clinical, treatment, and service-use related characteristics and the clinicians’ recommendation regarding continuity of care in a sample of young people who reached the upper age limit of their CAMHS. We found that the recommendation to continue treatment was primarily determined by clinician-reported severity of psychopathology and self- and parent-reported need for ongoing treatment, whereas the recommendation to continue treatment at AMHS rather than CAMHS was associated with treatment and service-use-related characteristics only, such as the length of CAMHS use and the availability of appropriate AMHS. In contrast with findings from some previous studies [6, 7, 17], an association between demographic and family characteristics and the recommendation to continue treatment at AMHS could not be confirmed. As both parental psychopathology [18] and not being in education, employment, or training are associated with negative mental health outcomes among young adults [19, 20], it is surprising that these characteristics are not considered in the clinician’s transition decision.

Clinician rated severity of psychopathology and self- and parent-reported need for ongoing treatment had the strongest associations with a recommendation to continue treatment. Although we expected clinician-reported problem level to be more strongly associated with the clinician’s recommendation to continue treatment than self- or parent-reported problem levels, due to shared source variance, the lack of an association between self- or parent-reported problem levels and the clinician’s transition recommendation was unexpected. As self- or parent-reported need for ongoing treatment was very strongly associated with the clinician’s recommendation, it might have suppressed the effects of self- and parent-reported problem levels in the multivariate model, resulting in non-significant effects. Even though self- and parent-reported problem levels were not associated with transition recommendations, our findings indicate that the need for ongoing treatment expressed by young people and parents are important factors in the transition decision. Fortunately, as the importance of including their perspective on the need for ongoing treatment has been emphasized by young people and parents [21, 22].

In contrast to findings from other studies [4, 6, 9, 17], having a clinical classification of a severe mental disorder did not increase the odds of being recommended to continue treatment. Suicidal thoughts/behaviours or self-harm, indicators of severe and acute problems, were not significantly associated with a recommendation to continue treatment either. Also, counterintuitively, two or more self-reported psychotic experiences were negatively associated with a recommendation to continue treatment. A possible explanation is that in a multivariate model with a strong general index for psychopathology, i.e., clinician-rated severity of psychopathology, the associations with other clinical markers for severity of psychopathology, such as having a severe mental disorder, suicidal thoughts/behaviours or self-harm and psychotic experiences, are suppressed. With regard to psychotic experiences, this explanation is supported by previous findings that self-reported psychotic experiences in the general population can be considered a clinical marker for severity of psychopathology [3, 23]. Although the presence of suicidality and psychotic experiences may be reflected in the clinicians’ assessment of severity of psychopathology, the additional explorative analyses showed that clinicians may not have been aware of the young person’s psychotic experiences in some cases, which may have affected their transition recommendations.

A clinical characteristic not studied previously was everyday functional skills, which were negatively associated with a recommendation to continue treatment. Young people with more everyday functional skills were more likely to be discharged or referred to other mental health services. Interestingly, whereas self-reported and parent-reported need for ongoing treatment were associated with the clinician’s recommendation to continue care, only parent-reported need for ongoing treatment was associated with the recommendation to continue treatment at AMHS. As parent-reported need for ongoing treatment was negatively associated with the recommendation to continue treatment at AMHS (and positively with continuing treatment in CAMHS), it may be that parent-reported need for ongoing treatment is associated with the involvement of parents in the young person’s treatment. As a systemic approach to treatment in which parents have an important role is more common in CAMHS than in AMHS [24], the involvement of parents may be a reason for the CAMHS clinician to continue treatment in CAMHS, rather than to refer the young person to AMHS. Alternatively, parents involved in the young person’s treatment may appeal to the CAMHS clinician to let the young person continue treatment in CAMHS, as they may be uncertain of what care in AMHS entails.

Treatment and service-use-related characteristics, such as psychotropic medication use and the length of service use within CAMHS, were specifically associated with a recommendation to AMHS. Findings from previous studies investigating these characteristics in relation to referral to AMHS have been inconsistent [4, 6, 7], due to differences in the operationalization of both medication use and length of CAMHS use, and differences in applied statistical analyses. It may be that psychotropic medication use and a longer length of treatment within CAMHS are indication of more severe and chronic psychopathology, requiring long-term treatment and a referral to AMHS. In some cases, the prescription and continuation of psychotropic medication might require contact with a specialist service such as an AMHS (e.g., the continuation of ADHD medication requires hospital contact at least once a year in France). Our findings also show that the CAMHS clinicians’ recommendation depends on their perspective on the availability of local AMHS services with the right skills and resources for some young people. Previous studies showed that CAMHS clinicians may not refer a young person to AMHS if they consider AMHS not to have the right expertise or will not accept certain referrals due to eligibility criteria [17, 25]. For instance, some AMHS may not accept referrals if they consider the young person to be ‘not ill enough’ [26] or eligibility criteria may exclude young people with specific diagnoses, such as neurodevelopmental disorders. Alternatively, clinicians may be unaware of the services offered by AMHS in the area, which may also affect the clinician’s transition decision.

### Strengths and limitations

This paper provides insight in the demographic and family, clinical and treatment, and service-use related characteristics that potentially play a role in the CAMHS clinician’s recommendation regarding continuity of treatment and transition to AMHS. The use of standardized assessments and young person and parent reports allowed us to reliably assess a large range of characteristics from the perspective of the young person, parent, and clinician. We conducted multivariate analyses with a large range of different predictors, accounting for most known potential confounders. There are several limitations to the findings reported in this study, as well. The limitations with regard to the recruitment process, representativeness, and generalizability were elaborately discussed in a paper describing the cohort profile [11] and will be discussed only briefly in this paper. First, a selection bias may exist due to several reasons: the selection of CAMHS participating in MILESTONE were not made randomly, but should be considered a convenience sample, the response rate was 45.1% (although other cohort studies on adolescents with mental health problems report similar response rates [27–29]) and the proportion of missing information, particularly among parents, is considerable. The response rate of 45.1% may be an overestimation of the true response rate that we would have obtained if information on the recruitment process was complete, but this estimate most accurately reflects the process. Due to medical ethical constraints, our recruitment methods relied on medical records and clinicians to assess eligibility and inclusion criteria, as well as to inform and gain consent from participants. Therefore, information on the recruitment process was incomplete. It was not possible to assess whether a selection bias exists with a non-response analysis, as the medical ethical committee did not allow us to collect data of non-responders without informed consent and very few young people consented to using this basic medical data. A previous analysis of missing data among participants [11] indicated a potential bias in participation of parents, who were less likely to complete all measures when their children reported more self-reported problems and clinicians reported more severe psychopathology. We applied multiple imputation to account for (a selection in) missingness in assessments. Ultimately, however, representativeness is not necessarily required to generalize our findings to other clinical populations of young people in the transition age [30]. Drawing reliable conclusions on the relationships between variables is possible if potential variables on which a selection could have taken place, such as parental educational level, country, gender, or severity of psychopathology are included in the analyses conducted, which was the case in the present study [31]. A selection bias to affect the generalizability of the findings from this study is therefore less likely. By recruiting young people from a wide range of CAMHS in different European countries, varying in size and ranging from community to specialist and/or academic hospital-based services in countries with differences in mental health service organization (i.e., more or less segmented services), culture, training, and concepts of mental health, our findings can be generalized independent of service type or European country. Replication of our study outside the European context is important to assess generalizability of our findings to other continents.

Finally, transition recommendations and need for ongoing treatment were assessed with a single item, part of the recently validated Transition Readiness and Appropriateness Measure [12]. To our knowledge, no other validated measures are available to assess these constructs. However, this could be considered a weakness of the study. Information on the clinician’s transition recommendations was missing for 16.1% of young people, but this is unlikely to impact the results of our study due to the application of multiple imputation.

### Conclusion and recommendations

In conclusion, the decision regarding continuity of treatment was most prominently determined by a small set of clinical characteristics, whereas none of these clinical characteristics determined whether transition to AMHS was recommended. Even though demographic and family characteristics may be important predictors of mental health outcomes, these characteristics are not considered in the clinician’s decision-making process. As the availability of appropriate AMHS was shown to be an important factor in the clinician’s transition decision, it is important that national and local governments guarantee AMHS availability and ensure that AMHS eligibility criteria meet the needs of young people with an ongoing need for treatment reaching the upper age limit of their CAMHS. Additionally, we recommend implementing standardized assessments of self- and parent-reported problems, including suicidality and psychotic experiences, in the period before young people reach the upper age limit of their CAMHS. Future analyses on MILESTONE cohort data will focus on the process of transition that follows the CAMHS clinician’s decision regarding transition, and will assess whether the clinician’s decision appropriately identifies young people who need ongoing treatment after reaching the upper age limit of CAMHS. Future analyses will also show which characteristics are associated with actually making a transition to AMHS or, alternatively, experiencing an unplanned discontinuity of treatment, as well as associated mental health outcomes.

## Supplementary Information

Below is the link to the electronic supplementary material.

Supplementary file1 (PDF 221 KB)

## Data Availability

The participant consent forms restrict data sharing on a public repository. The MILESTONE consortium invites researchers to contact the corresponding author for requests for statistical code used, instruments used, and anonymised data.

## Code availability

All analyses were conducted in R. Code can be made available upon request to the corresponding author.
