# Supplementary material for: Demographic, clinical, and service-use characteristics related to the clinician’s recommendation to transition from child to adult mental health services
Source: Soc Psychiatry Psychiatr Epidemiol. 2022 Feb 10;57(5):973–91. doi: 10.1007/s00127-022-02238-6 (PMC9042957; doi:10.1007/s00127-022-02238-6)

Article: Demographic, Clinical and Service-use Characteristics related to the Clinician's Recommendation to Transition from Child to Adult Mental Health Services

Journal: Social Psychiatry and Psychiatric Epidemiology

Authors: S. E. Gerritsen[1] & L. S. van Bodegom[2,1], G. C. Dieleman[1;1], M. M. Overbeek[2,3], F. C. Verhulst[1,4], D. Wolke[5], D. Rizopoulos[6], R. Appleton[7], T. A. M. J. van Amelsvoort[8,9], C. Bodier Rethore[10], F. Bonnet-Brilhault[10], I. Charvin[11], D. Da Fonseca[11], N. Davidović[12,13], K. Dodig-Ćurković[14,15,16], A. Ferrari[17,18], F. Fiori[19,20,21], T. Franić[12,13], C. Gatherer[22], G. de Girolamo[17], N. Heaney[19], G. Hendrickx[23], R. Jardri[24], A. Kolozsvari[21], H. Lida-Pulik[25], K. Lievesley[19], J. Madan[26], M. Mastroianni[19,20], V. Maurice[27], F. McNicholas[28,29], R. Nacinovich[30,31], A. Parenti[32], M. Paul[22,33], D. Purper-Ouakil[27,34], L. Rivolta[35,36], V. de Roeck[37,38], F. Russet[27], M. C. Saam[39], I. Sagar-Ouriaghli[19], P. J. Santosh[19,20,21], A. Sartor[40], U. M. E. Schulze[39], P. Scocco[41,42], G. Signorini[17], S. P. Singh[22], J. Singh[19,20], M. Speranza[43,34], P. Stagi[44], P. Stagni[17,45], C. Street[22], P. Tah[22], E. Tanase[46], S. Tremmery[37], A. Tuffrey[22], H. Tuomainen[22], L. Walker[22], A. Wilson[22], A. Maras[2,1] for the Milestone consortium.

Affiliations: [1]Department of Child and Adolescent Psychiatry and Psychology, Erasmus Medical Center, Rotterdam, Netherlands, [2]Yulius Academy, Yulius Mental Health Organization, Dordrecht, Netherlands, [3]Clinical Child and Family Studies, Vrije Universiteit Amsterdam, Amsterdam, the Netherlands, [4]Department of Clinical Medicine, University of Copenhagen, Copenhagen, Denmark, [5]Department of Psychology, University of Warwick, Coventry, United Kingdom, [6]Department of Biostatistics, Erasmus Medical Center, Rotterdam, The Netherlands., [7]NIHR Mental Health Policy Research Unit, Division of Psychiatry, University College London, London, United Kingdom, [8]Department of Psychiatry and Neuropsychology, University of Maastricht, Maastricht, The Netherlands, [9]Mondriaan Mental Health Care, Heerlen, the Netherlands, [10]Centre Hospitalier Universitaire de Tours, France, [11]Centre Hospitalier Universitaire de Marseille, France, [12]University Hospital Split, Split, Croatia, [13]School of Medicine, University of Split, Croatia, [14]Faculty for Dental Care and Health, Osijek, Croatia, [15]University Health Center Osijek, Osijek, Croatia, [16]Unit for Child and Adolescent psychiatry, Osijek, Croatia, [17]IRCCS Istituto Centro San Giovanni di Dio Fatebenefratelli, Brescia, Italy, [18]DISM, ULSS 16, SOPROXI Onlus, Padua, Italy, [19]Department of Child & Adolescent Psychiatry, Institute of Psychiatry, Psychology and Neuroscience, Kings College London, London, United Kingdom, [20]Centre for Interventional Paediatric Psychopharmacology and Rare Diseases, South London and Maudsley NHS Foundation Trust, London, United Kingdom, [21]HealthTracker Ltd, Kent, United Kingdom, [22]Warwick Medical School, University of Warwick, Coventry, United Kingdom, [23]Department of Neurosciences, Centre for Clinical Psychiatry, KU Leuven, Leuven, Belgium, [24]Université de Lille, INSERM (U-1172), Lille Neurosciences & Cognitions, Plasticity & SubjectivitY team, CURE platform, Fontan Hospital, CHU Lille, France, [25]CH Versailles, Versailles, France, [26]Warwick Clinical Trials Unit, Warwick Medical School, University of Warwick, Coventry, United Kingdom, [27]Centre Hospitalier Universitaire de Montpellier, Saint Eloi Hospital, Montpellier, France, [28]School of Medicine & Medical Science, University College Dublin, Dublin, Republic of Ireland, [29]Lucena CAMHS, SJOG, Dublin, Republic of Ireland, [30]Child and Adolescent Neuropsychiatry Unit, ASST Monza, Italy, [31]Università degli Studi Milano Bicocca, Milano, Italy, [32]Centre Hospitalier Universitaire de Lille, France, [33]Coventry and Warwickshire Partnership NHS Trust, Coventry, United Kingdom, [34]INSERM, CESP U1018, PsyDev, University Paris Saclay, UVSQ, Versailles, France, [35]Psychiatric Epidemiology and Evaluation Unit, Saint John of God Clinical Research Center, Brescia, Italy, [36]Department of Mental Health, Psychiatry Unit, San Gerardo Hospital, Monza Brianza, Italy, [37]Department of Neurosciences, KU Leuven, Leuven, Belgium, [38]Child and Youth Studies, Campus Social School, University Colleges Leuven Limburg, Heverlee, Belgium, [39]Department of Child and Adolescent Psychiatry/Psychotherapy, University of Ulm, Ulm, Germany, [40]Josefinum Augsburg, Klinik für Kinder- und Jugenspsychiatrie und Psychotherapie, Augsburg, Germany, [41]Department of Mental Health, ULSS 6 Euganea, Padua, Italy, [42]SOPROXI Onlus, Padua, Italy, [43]Service Universitaire de Psychiatrie de l'Enfant et de l'Adolescent, Centre Hospitalier de Versailles, France, [44]Child and Adolescent Neuropsychiatry Unit, AUSL Modena, Italy, [45]Child and Adolescent Neuropsychiatry, Department of Mental Health, Modena, Italy, [46]ZfP Südwürttemberg, Abteilung für Psychiatrie und Psychotherapie des Kindes-und Jugendalters Weissenau, Ravensburg, Germany

Corresponding author: Dr. Gwen Dieleman, Department of Child and Adolescent Psychiatry/Psychology, Erasmus Medical Center. P.O. Box 2060, 3000 CB, Rotterdam, the Netherlands. g.dieleman@erasmusmc.nl, +31107038196.

Supplementary Fig. 1 – Effect plot presenting the effect of clinician-rated severity of psychopathology on the clinician's recommendation to continue care

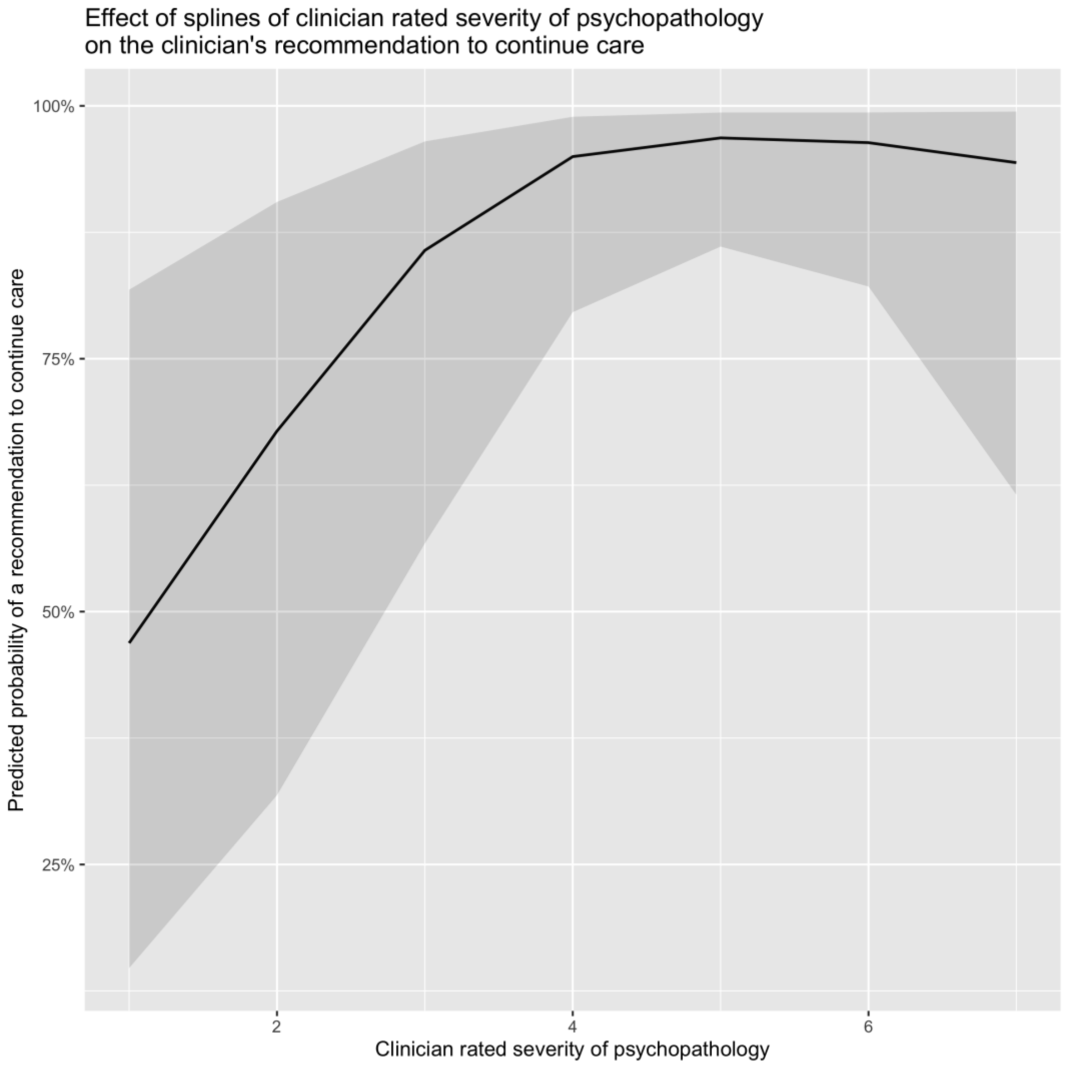

Supplement: Supplementary file 1 — Supplementary file1 (PDF 221 KB) [file 127_2022_2238_MOESM1_ESM.pdf]
